# Pathway(s) and distribution of photosystem II-cyclic electron flow

**DOI:** 10.1042/BST20250531

**Published:** 2026-10-06

**Authors:** Grant Steiner, Chloe Wilke, Colin Gates

**Affiliations:** 1 Department of Chemistry and Biochemistry, Loyola University Chicago, 1068 W. Sheridan Road, Chicago, IL 60660, U.S.A.; 2 Department of Bioinformatics, Loyola University Chicago, 1032 W. Sheridan Road, Chicago, IL 60660, U.S.A.

**Keywords:** biotechnology, photoprotection, photosystems, plastoquinone, water oxidizing complex

## Abstract

Photosystem II is a highly conserved protein complex found across all oxygenic phototrophs and the source of almost all biologically available electrons and atmospheric oxygen. This large, membrane-bound complex is at continuous risk of damage under direct, full sunlight, and those organisms that live under such conditions have developed photoprotective mechanisms to address this stressor. One such mechanism, photosystem II-cyclic electron flow (PSII-CEF), is a poorly understood pathway by which charge-separated electrons are recombined with holes after an extended separation and multiple electron transfer events. This mechanism is critical to the survival of the fastest-growing known eukaryotic phototrophs, playing a critical role in both native extremophiles and lab-adapted strains. However, the path electrons take during this recombinative process is not known. In the present review, we address the state of the field and the challenges facing implementation of PSII-CEF as a component of photosynthetic organism protection, survival, and improved growth.

## The photosynthetic apparatus

Phototrophic organisms across the tree of life utilize photosynthetic electron transfer (ET) as a means of sourcing chemical energy from solar radiation. This electron transport chain operates differentially under dynamic environmental conditions in virtually every habitat around the globe [1–3]. Commonly, however, when one discusses ‘photosynthesis’ as a biochemical mechanism, they are generally referencing linear electron flow (LEF). In oxygenic phototrophs, electrons are stripped from water and sequentially transferred to several redox-active intermediates, the last of which is NADP^+^ [4,5]. These electrons traverse three protein supercomplexes in the thylakoid membrane of oxygenic phototrophs. These proteins, photosystem II (PSII) [6,7], cytochrome (cyt) *b*_6_*f* [8,9], and photosystem I (PSI) [10,11], work in this order in LEF. ET between the major protein complexes in LEF is bridged by the two-electron redox carrier plastoquinone (PQ) from PSII to cyt *b*_6_*f* and the one-electron carriers plastocyanin (PC) or cyt c_6_ from cyt *b*_6_*f* to PSI [12–14].

The reduction of NADP^+^ to NADPH sequesters the energy transduced from photon acquisition in the photosystems [15,16]. Photosynthetic electron transport chain (PETC) operation also produces a proton gradient across the thylakoid membrane, which powers the phosphorylation of ADP to ATP on the stromal side of the membrane by ATP synthase [17,18]. Ultimately, the NADPH and ATP produced via the electrons and protons removed from water are later expended in carbon fixation during the Calvin–Benson–Bassham cycle [19,20]. Assuming efficient linear ET through the whole chain, including the Q-cycle between PSII and cyt *b*_6_*f*, the transfer of four electrons from two molecules of water will deposit 12 protons within the thylakoid lumen, driving the synthesis of two molecules of NADPH and either 2 or 3 ATP (on average 12/14 × 3 = 2.57), for an ATP:NADPH ratio of 1.28 [21–23]. This ratio comes close to the energy needed to fuel the Calvin–Benson–Bassham cycle (1.5 ATP:NADPH) [19,20,24], but only under the assumptions that (1) the PETC operates linearly with perfect efficiency, and (2) respiration creates the ATP that fuels every other cellular process catalyzed by phosphorylation. In practice, this is often not the case, and cyclic electron flow around PSI recycles these electrons from after PSI into the PQ pool, performing a separate Q-cycle and increasing the number of protons generated per water oxidized [25]. A dynamic need for increasing the ATP:NADPH ratio to continue carbon fixation is thought to be a driving force behind employing alternative routes of ET in oxygenic phototrophs [26–28].

## Photosystem II: nature’s water oxidation enzyme

Molecular oxygen production by oxygenic phototrophs has shaped the atmosphere [29–31] and constrained the evolution of life since the ‘Great Oxygenation Event’ ca. 2.4 Ga [32,33]. These chemical and ecological changes owing to the exponential increase in O_2_ production are the result of the first protein complex in the PETC: PSII. To date, PSII is the only known naturally occurring enzyme able to oxidize water. Structurally, a single monomer of PSII is a complex of more than 20 subunits, with the specific number depending on the organism and environmental conditions [6,7,34,35]. Typically, two fully intact monomers of PSII will form a dimer through linker proteins. A functional PSII monomer consists of a core, extrinsic proteins that protect the water-oxidizing complex (WOC), and varying low molecular mass proteins (<10 kDa). A functional unit of PSII typically associates with a light harvesting complex, which exhibits a variety of structures depending on the organism [36–38]. This complex works as an antenna to broaden the wavelength and spatial range of light that can be captured for photosynthesis. PSII monomers each contain several dozen cofactors, including 35 chlorophylls (chl), 10 to 12 β-carotene, 2 pheophytins (pheo), a non-heme iron (NHI), at least 2 PQs, a CaMn_4_O_5_ cluster (WOC), and various lipids and ions [39–42]. The core composition of PSII is highly conserved across oxygenic phototrophs (Figure 1).

**Figure 1:**
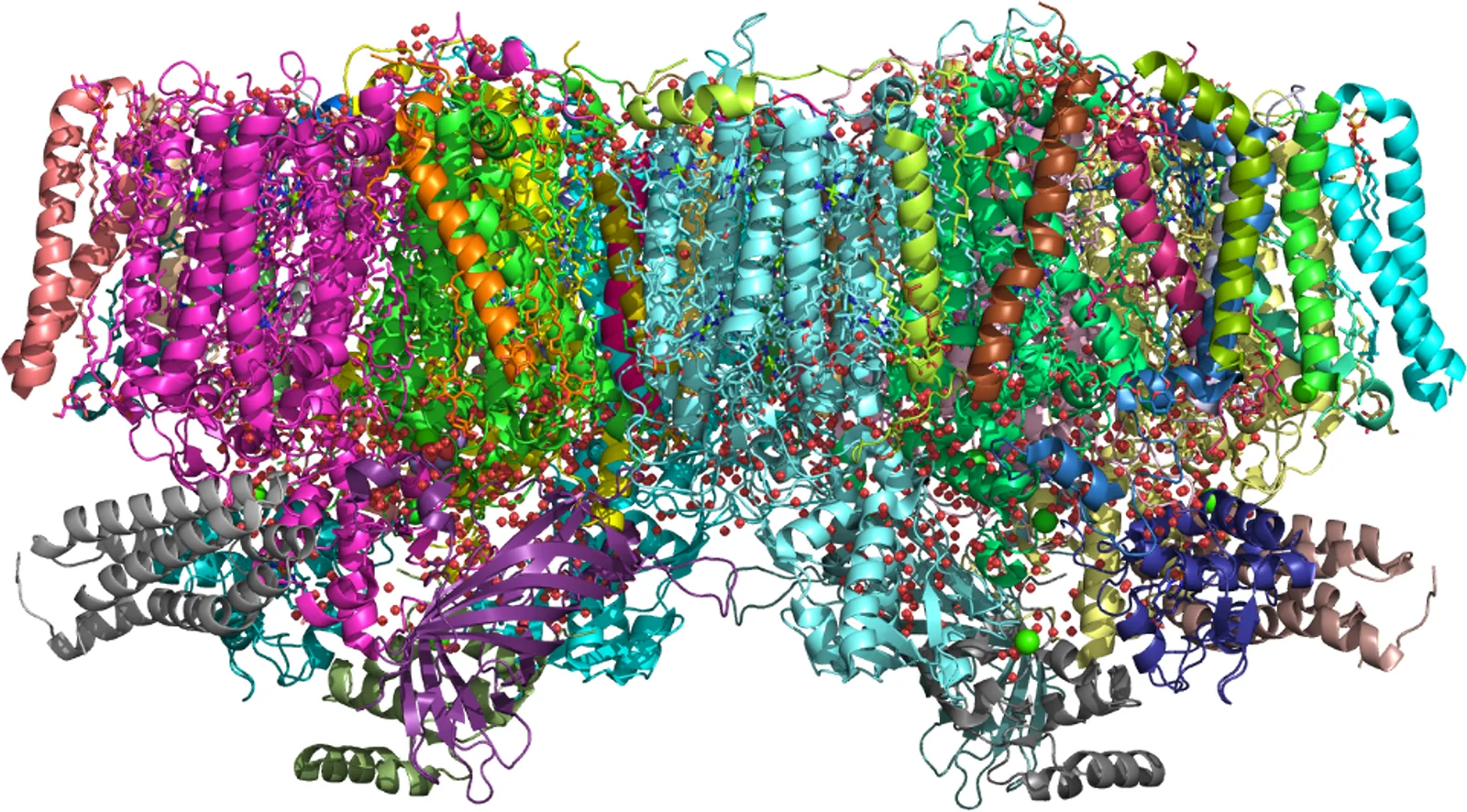
PSII–protein complex 1.93 Å resolution cryo-EM structure of a PSII dimer from the cyanobacterium *Synechocystis* sp. PCC 6803. [PDB ID: 7N8O] [41].

The reaction center (RC) of PSII is a pseudo-homodimer encased by PsbA and PsbD, also known as the D1 and D2 subunits. These subunits are the most central in PSII and coordinate special chl, the farthest-red Chl_D1_ and Chl_D2_, and the photochemically active P_D1_ and P_D2_, which form a tetramer at the heart of PSII, called P680 for the red-shifted chl absorption peak of the center at 680 nm. PsbB and PsbC are integral core antenna proteins, also known as CP47 and CP43, respectively, for chl proteins with apparent weights of 47 and 43 kDa [43]. PsbE and PsbF are the alpha and beta subunits of cyt *b*_559_, a redox-active cofactor necessary for the assembly of PSII and successful water oxidation [44–46], but the involvement of cyt *b*_559_ in the process of ET through PSII is still ambiguous (and will be expanded upon subsequently). Each of these core subunits works cooperatively to ensure the enzymatic activity of the protein complex. Extensive literature showcases that knockouts of any individual component or active site mutagenesis are detrimental to PSII operation [44,47,48].

While the architecture of the PSII core is tightly regulated, flexibility to modify excitation energy delivery to the RC is critical to avoid inactivation of the D1 subunit. When a photon reaches the RC, a charge separation event results in the transfer of the excited electron transiently to a pheo molecule, then on to the first acceptor PQ, Q_A_ [49–52]. Both of these processes are only possible if each acceptor is oxidized when charge separation occurs. The resulting electron hole is initially localized on P_D1_ and P_D2_ in the RC [53,54]. This P680^+^ has an extreme oxidizing potential (∼1.3 V), driving the removal of an electron from a nearby specialized tyrosine residue, D1-Y161 (or Y_Z_). Y_Z_ can then sequentially remove electrons from nearby Mn atoms housed in the WOC, which cycles through four individual and increasing oxidation states [55–58]. The terminal electron acceptor in PSII is a reversibly bound PQ, Q_B_, which will sequentially accept electrons from Q_A_ past a NHI cofactor complexed to a bicarbonate molecule [59]. Unlike Q_B_, Q_A_ does not undergo protonation and is not exchangeable in its binding site (binding energy of −56.1 kcal/mol) [60,61]. As a result, Q_A_ is only able to transfer a single electron to Q_B_ at a time. After being reduced to plastoquinol (PQH_2_), Q_B_ will carry electrons forward through the PETC by exchanging out of the site. An illustration of the cofactors involved in LEF (and prospective photosystem II-cyclic electron flow (PSII-CEF) participants) is available below as Figure 2.

**Figure 2:**
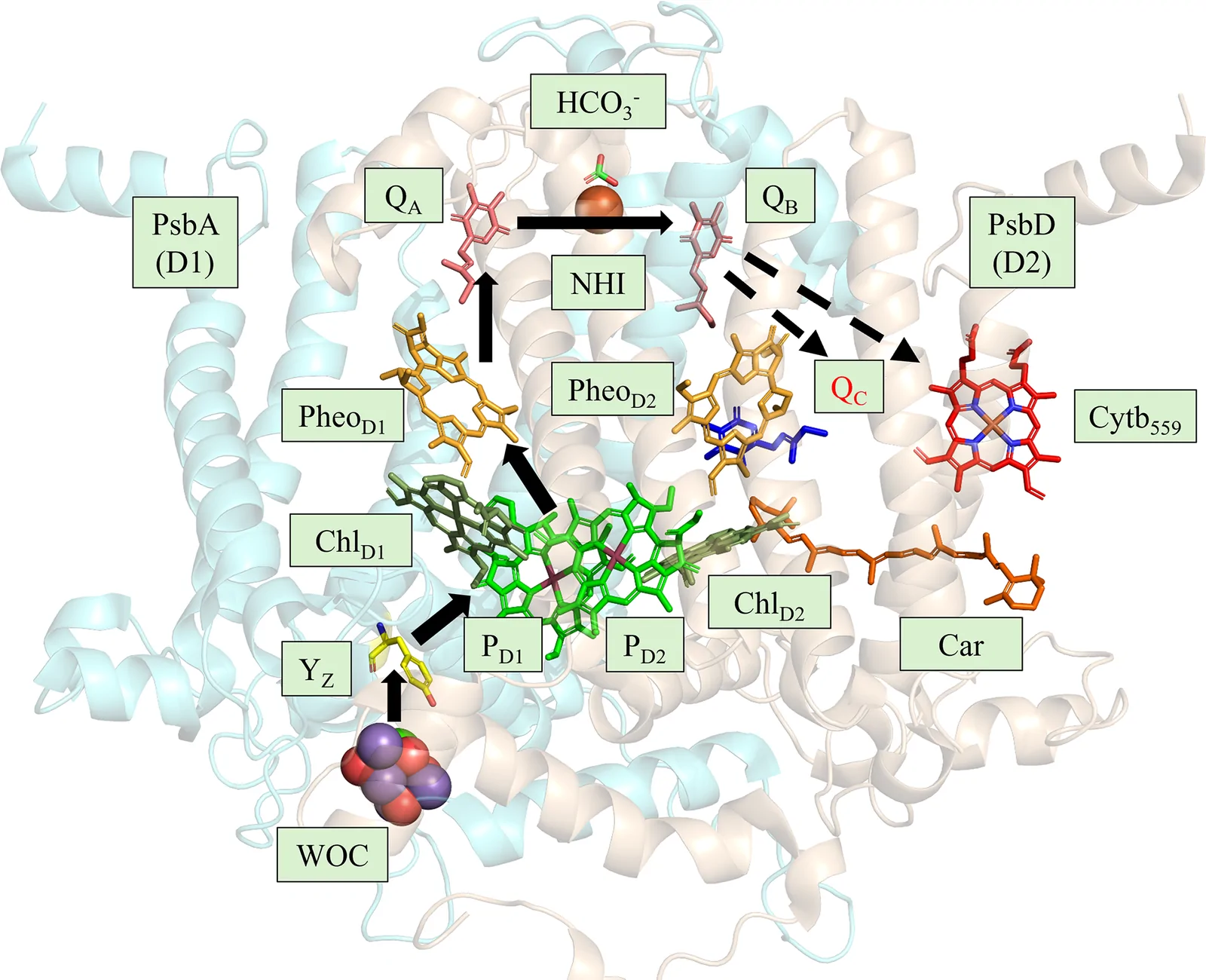
Illustration of the LEF cofactors in the core of PSII Structural representation of the cofactors in LEF from the WOC to Q_B_, as well as the D2 branch cofactors and putative PSII-CEF cofactors. The illustration is a modification of the PDB structure 7N8O; hydrocarbon tails have been removed from all chl, pheo, and quinones. Q_C_ site is inferred from PDB structure 4V62.

Conversely, if a build-up of reductant on the acceptor side of PSII is present during an attempted charge separation event and pheo cannot be reduced, there are multiple possible outcomes. If the exciton recombines into the open electron hole, the absorbed photon can either be emitted as fluorescence or undergo a dissipation event to accessory pigments in PSII, which will remove the excess energy as heat [62,63]. This heat dissipation process in PSII is termed non-photochemical quenching (NPQ), as the energy is quenched without the occurrence of photochemical activity [64,65]. With increasing light, a third possibility arises whereby the excited chl is further promoted to a long-lived triplet spin state that may interact with ubiquitous O_2_ to create reactive singlet oxygen [66–68]. These reactive oxygen species may indiscriminately interact with residues in PSII and cause irreparable damage. This process of ‘photoinhibition’ occurs increasingly at higher intensities of light, necessitating protection of LEF in organisms under dynamic light conditions.

## Early literature history of PSII-CEF

One posited way that phototrophs work to protect PSII in high light conditions is by cycling electrons removed from the acceptor side of the enzyme back to the donor side in a process currently termed PSII-CEF. Alternatives to the conventional linear pathway have been investigated for decades, with the reduction of cyt *b*_559_ dating to the work of Whitmarsh and Cramer in 1977 [69]. The earliest demonstrations of a photoprotective mechanism were published in 1979, showing that electron flow from water to P680 could be inhibited while maintaining electron acceptance from the RC [70,71]. The use of uncoupling agents in early work demonstrated that changes to the redox potential of cyt *b*_559_ occur when donor side ET is affected or when light intensities are increased [71,72]. Although these findings all suggested internal modes of ET through PSII, external carriers such as PQ [73] and PC [74] were soon suggested to return electrons to P680^+^.

The first whole cell study without the use of chemical inhibitors came about in 1986, when the group of Paul Falkowski investigated a green alga, *Chlorella pyrenoidosa* [75]. This group found that in increasing continuous light conditions, variable fluorescence (F_V_), commonly used as a metric of PSII RC operation quantity, remained relatively constant while the oxygen yield diminished over time. Here, it had been shown that electrons were leaving P680 at a consistent rate, but the holes were being quenched by something other than the oxygen evolution of the Joliot–Kok cycle. Over a decade later, the first potential mechanism for this alternative flow was proposed. Studies in *Spinacia oleracea* showed that the chemical 4-[methoxybis(trifluoromethyl)methyl]-2,6-dinitrophenylhydrazinemethyl ketone (K-15) could accept electrons directly from pheo and shuttle them to cyt *b*_559_ to avoid photobleaching [76]. The proposed mechanism then suggested ET through an accessory chl to Y_Z_.

Multiple works by Miyake et al. [77–79] determined comparisons in the functioning of internal PSII-CEF to NPQ in spinach chloroplasts, showing that internal ET through PSII is ΔpH-dependent, but not in the same ways that NPQ is. These studies also helped to corroborate the hypothesis that PSII-CEF works as a photoprotective mechanism, as inhibiting ET through PSII degrades its activity but does not change ET through PSI. In 2006, more holistic evidence for the occurrence of PSII-CEF came in the form of increasingly mismatched rates of carbon fixation to oxygen production in sunflower and tobacco leaves tested under saturating light conditions [80]. In the same year, electron paramagnetic resonance spectroscopy on the diatom *Phaeodactylum tricornutum* showed a lack of involvement of Y_D_, a tyrosine residue present in the D2 subunit and analogous to Y_Z_, in the PSII-CEF mechanism [81]. This same study found that *P. tricornutum* employs a combination of PSII-CEF and NPQ to hinder photoinhibition.

Major strides in the understanding of PSII processes came about a few years later as newly available crystal structures, mainly from cyanobacterial systems, garnered a sizable interest in the photophysiology of this complex [40,82,83]. Up to this point, there had not been a consistently utilized model to study the processes of PSII-CEF, making justification of potential pathways by direct comparison to available protein structures difficult. Mechanistically, all that had seemed consistently clear was that a disparity between rates in charge separation and oxygen evolution helped reduce photoinhibition at increasing light intensities, which was not a conserved feature across all phototrophs. It would stand to reason that there is a requirement of evolutionary pressure to induce the structural changes responsible for this photoprotection.

## Cytochrome *b*_559_

One of the earliest suggested modulators of an internal PSII-CEF mechanism is the peripheral cofactor cyt *b*_559_. Cyt *b*_559_ consists of a heme group, ligated to a histidine residue on each of the α and β subunits, PsbE and PsbF. Spatially, cyt *b*_559_ is adjacent to the D2 subunit, lying outside the region of redox intermediates directly involved in LEF. Despite this, various studies have exhibited that the redox poise of cyt *b*_559_ is dynamic and has direct effects on the efficiency of photochemical quenching and acts as a PQ oxidase in a multitude of phototrophs [84–87]. The heme group of this complex exhibits a range of redox potentials with poorly understood distinction in structure between them, but postulated differences in function. The low potential (LP, ∼0 mV) form is more easily induced experimentally than the intermediate potential (IP, ∼200 mV) or high potential (HP, ∼390 mV) forms [88,89].

It is believed that switching between these redox potentials helps to modulate the mode of electron flow through or around PSII. In terms of induction by high light intensity, the LP form becomes prevalent and is thought to oxidize Q_A_^−^ when there is reductant stress to the acceptor side of PSII. Conversely, the HP form is thought to respond to donor side stress, donating to the RC when the WOC or Y_Z_ are unable to. Original characterization of the photooxidation kinetics in cyt *b*_559_ by electron paramagnetic resonance [90], near-infrared absorbance [91], and shifted-excitation Raman difference spectroscopy [92,93] showcased that the heme group equilibrates an oxidizing equivalent with a nearby accessory chl and carotenoid, suggesting ET through each of these pigments in a cyclic pathway proposed by the group of Brudvig [94,95] and expanded by them over the last three decades [89,96,97]. Having the lowest potential in this redox-active triad, cyt *b*_559_ likely reduces the accessory pigments, but it is not clear whether the chl or carotenoid directly transfer electrons to P680. The other uncertainty inherent in this proposed pathway is the direct reduction of cyt *b*_559_ by Q_B_ which is an approximate distance of 25 Å away. This distance and potential gap would not be able to facilitate timescales fast enough to explain experimental studies in chloroplasts and whole cells that would require ET kinetics on the order of ms; cornerstone work on this subject found sub-Hz frequencies of reoxidation [96]. For this to be achievable, there likely needs to be an intermediate cofactor between the two.

## Acceptor side quinone(s)

Currently, there are two main proposed pathways for PSII-CEF: an internal single-electron mechanism and an external two-electron exchange through the PQ pool [98]. One of the few conserved features of each proposed pathway or regulatory mechanism of PSII-CEF is that electrons at least reach the PQ in the Q_B_ site. The midpoint potentials (E_m_) of the Q_B_/Q_B_^•−^ and Q_B_^•−^/Q_B_H_2_ pairs are 90 and 40 mV, respectively [99], with the Q_B_^•−^ semiquinone thermodynamically stabilizing the PQ, resulting in a higher E_m_ and preventing backward electron movement. For internal one-electron CEF, the electrons need to be transferred from Q_B_ back to either the WOC, Y_Z_, or P680^+^ within the RC. There have been multiple proposed pathways for different redox sites involved in this transfer, the most common of which is cyt b_559_. Whether these electrons are directly brought to the donor side, shuttled to an internal redox carrier, brought forward in the chain and returned later, or even which donor side structure the electrons are returned to, all remain ambiguous.

In the cyanobacterium *Thermosynechococcus vulcanus*, a putative third PQ binding site, Q_C_, was observed lumenal to Q_B_ (i.e., near the middle of the thylakoid membrane), with an approximate distance of 12 Å between the quinone head groups [100,101]. The prevalence of the Q_C_ site appears to be dependent on preparation methods [40], likely due to the lower binding affinity of the polar quinone head group compared with Q_B_ [60]. While Q_A_ is found 13 Å from Q_B_, allowing for the fast redox transfer between these key sites [102], there is a larger distance between Q_B_ and cyt *b*_559_ (25 Å) [89], strongly implying that an intermediate redox-active site is required for electron flow between these distant carriers. In this sense, if a Q_C_ quinone is found in PSII, it may work effectively as a bridging intermediate, but its more common absence in PSII structures effectively hinders understanding of the PSII-CEF internal mechanism and whether it passes through this site. Due to this, there is limited understanding of the involvement or necessity of Q_C_ in PSII. A study on the effects of several point mutations to cyt b_559_ and PsbJ (an α helix that tunes the E_m_ of Q_B_/Q_B_^−^) [103] near key Q_C_ interactions found that there were no significant changes to the oxygen production, PQ pool redox poise, or PSII content in any of the mutants, but significant changes to NPQ levels were observed [104,105].

## State of the field

The extant literature surrounding the mechanism(s) of PSII-CEF has recently been expanded to studies by pulsed chl *a* fluorescence. This technique, termed fast repetition rate (FRR) fluorometry [106], has been utilized in combination with a Joliot–Kok model-derived energy-minimization matrix model, known as VZAD [107], to model electronic cycling of the WOC in several publications [108–111], many of which have been utilized to study the occurrence of electron return to the WOC specifically. VZAD is able to measure inefficiency parameters in the period-four oscillation of the WOC and the period-two oscillatory behavior of the acceptor quinones of PSII, causing an equilibration of S-states over a series of flashes. By monitoring how these inefficiencies affect F_v_, the group of Charles Dismukes has observed that electron return to the WOC only occurs in states of PSII that possess Q_B_^•−^ prior to a saturating flash of light. This flash creates a state of Q_A_Q_B_^2−^, which can redirect electrons back to the WOC, but only when it is in an S-state that is not dark stable, namely either the S_2_ or S_3_ states [108]. This electron return reaches a maximum rate when there is a moderate ET rate through cyt *b_6_f* (∼0.03 s^−1^), and seems to be directly tied to the redox poise of the PQ pool, which will likely become a central point of modulation for regulating the rates of PSII-CEF in newly engineered photosynthetic microbes.

For the most part, PSII-CEF has been observed or extrapolated from indirect experimental studies, and without a clear mechanism there has been limited effort toward intentional use of the mechanism for the improvement of crops. As PSII-CEF appears to be primarily a photoprotective mechanism that facilitates desiccation tolerance, it can be found in organisms that encounter extreme light or low water in their native environments [112,113]. It is specifically not a major contributor in some organisms that are highly halotolerant, such as *Limnospira maxima* [111,112] and the genus *Picochlorum* [114], even though these organisms experience high light and osmotic stress. The extent of PSII-CEF across the tree of life remains unknown, which means that those clades in which it has already been observed are the easiest current targets for induction of the process in organisms not currently making use of it.

In 2013, a microalga isolated from an arid biological soil crust in the Negev Desert, Israel, garnered significant research interest for its extremophilic capabilities [115]. This alga, named *Chlorella ohadii* in honor of Itzhak Ohad, can survive severe drought conditions and light intensities up to 3500 μmol photons m^−2^ s^−1^, 150% of the maximum illumination reached in its natural habitat [113,115–117]. This extraordinary resistance results in *C. ohadii* achieving some of the highest photosynthetic activity and growth rates on record under extremely high light. Flash oximetry studies revealed that this PETC operation included over 90% of electrons separated by PSII being returned to P680 by PSII-CEF [113]. Unlike what had previously been seen in *P. tricornutum*, *C. ohadii* does not express stress-response proteins necessary for NPQ, suggesting its PSII photoprotection is almost entirely from electron recycling [117–119]. The robust response of *C. ohadii* against environmental stressors has since led to considerable investigation into its photophysiology, structures of photosystems, and mechanisms of metabolism. While several organisms have contributed to our current understanding of PSII-CEF, *C. ohadii* is a critical model of investigation as the most active known user of the mechanism. As it can advance 90% of electrons at 2000 μmol photons m^−2^ s^−1^ via PSII-CEF, this requires a turnover rate on the order of hundreds of Hz, close to the turnover rate limit of the WOC [113].

As *C. ohadii* is the most active known user of PSII-CEF as a component of its photoprotection strategy, its clade represents a good starting point for strain selection. The genus *Chlorella* and the many genera that have been split from it are commonly used as bioproduction organisms [120,121], meaning infrastructure already exists to test and grow strains selected for PSII-CEF use. The currently unknown genetic machinery that supports PSII-CEF is present across some subset of this clade. While *Picochlorum* appears to use more conventional mechanisms such as NPQ [113], within *Chlorella* other species are capable of using PSII-CEF. We demonstrated this using a *Chlorella vulgaris* strain that had been in a culture collection for 35 years [122], and thus selected for growth under constant dim light and water availability, following collection from a low-light, temperate environment. This strain was subjected to selective pressure for survival of increasingly high light levels in liquid culture under high carbon dioxide and stable temperature, constraining the stress to specifically be excess light. As a result of dozens of generations of pressure, eventually reaching 2000 μmol photons m^−2^ s^−1^, the lineage became far more robust, decreasing its doubling time from 1.8 days to a minimum of 1.6 hours. It additionally was able to survive and grow, albeit more slowly, without supplemental CO_2_.

Subsequent research currently in press resulted in similar behavior in *Chlorella sorokiniana*, the closest relative of *C. ohadii* [123]. In these organisms, however, various warning signs emerge. While *C. ohadii* is able to preserve some level of FRR-observable WOC cycling at extreme light, suggesting spatial separation of PSII for different purposes [110], its acclimated relatives undergo generalized recombination and the WOC cycle is only observable after addition of a supplementary electron acceptor [122]. While this is the hallmark of PSII-CEF in flash oximetry [112], it is only observable by addition of an exogenous electron acceptor, which constrains observation of the process. A further difference in PSII behavior is antenna management in *C. ohadii*, which can minimize its antenna to the point of being essentially nonexistent in some studies and undergo extremely rapid state transitions as compared with any other examined phototrophs [113]. Despite the fact that the three *Chlorella* strains have different survival mechanisms for these light stress conditions, all perform about the same amount of LEF per PSII, reaching one electron successfully removed from oxygen every 13–14 ms on average under growth light. This suggests that the more critical determinant, as long as PSII-CEF is available, is whether this process, state transitions/antenna management, NPQ, or some other mechanism such as a terminal oxidase-based water-water cycle [124] is most beneficial to survival. The basis behind such a selection remains to be determined.

As PSII-CEF is best developed in microalgae, until it can be more clearly observed in other phototrophs, especially crop plants, its practical applications are most applicable in the fields where microalgae are actively being investigated—biofuel production and energy sustainability [125]. Combining biophysical measurements with genetic engineering will allow for the study of key photoprotective pathways and the future design of resilient phototrophs for energy, instrumentation development, and agricultural applications [126]. Photoprotective mechanisms in algae and cyanobacteria, such as the induction of PSII-CEF, can be harnessed to modify crop plants to withstand harsh environmental conditions due to the highly conserved nature of PSII across photosynthetic organisms. There are generally more electrons produced by PSII than consumed in carbon fixation even under ideal circumstances, resulting in unused energy. Understanding and developing redox control in these coupled pathways can result in higher energy yields under stress environments. Accordingly, further investigation and development of both algal processes and plant analogs is a critical direction for future research.

## Perspectives

- As atmospheric carbon dioxide levels increase and hot, dry climates cover an increasingly large area of the globe, it is necessary to investigate methods by which current or future crop organisms may survive these changing conditions.
- PSII-CEF bears clear benefits to those organisms that make extensive use of it, but the mechanism by which it is carried out remains poorly understood. Furthermore, the extent of PSII-CEF and the range of organisms that are capable of utilizing this process have not been fully explored.
- Selecting for elevated PSII-CEF, as currently understood, is selecting for survival of common field stressors. The mechanisms that allow fast-growing phototrophs to survive harsh environments will have translational applications.

## Data Availability

No new data were collected or processed for the creation of the present review.

## Open Access

Open access for this article is provided via our Subscribe to Open programme, made possible through the support of our subscribing institutions. Learn more here: https://portlandpress.com/pages/open_access_options_and_prices#conditional.

## Abbreviations

chl: chlorophyll
cyt: cytochrome
ET: electron transfer
LEF: linear electron flow
F_V_: variable fluorescence
HP: high potential
IP: intermediate potential
LP: low potential
NHI: non-heme iron
NPQ: non-photochemical quenching
PC: plastocyanin
PETC: photosynthetic electron transport chain
pheo: pheophytin
PSI: photosystem I
PSII: photosystem II
PSII-CEF: photosystem II-cyclic electron flow
PQ: plastoquinone
RC: reaction center
WOC: water-oxidizing complex
